# Dietary simple sugar intake, metabolic indicators, markers of inflammation, and injury among semi-professional football players

**DOI:** 10.29219/fnr.v69.11036

**Published:** 2025-01-27

**Authors:** Jun Hou, Yuemei Cui, Jun Gao, Ming Rong

**Affiliations:** 1 Faculty of Sports Science, Ningbo University, China; 2 Research Academy of Grand Health, Ningbo University, Ningbo, China; 3 Institute of Sports Medicine and Health, Chengdu Sport University, Chengdu, China

**Keywords:** simple sugar, glucose, fructose, inflammation, high-sensitivity C-reactive protein, adenosine deaminase

## Abstract

**Background:**

Dietary sugar intake has been implicated in the development of metabolic dysfunction, chronic inflammation, and immune dysfunction, contributing to the pathogenesis of various diseases. This study aimed to investigate the associations between dietary total simple sugar intake and glycemic markers, lipid profile, serum levels of high-sensitivity C-reactive protein (hs-CRP), and adenosine deaminase activity (ADA), among semi-professional football players.

**Methods:**

A cross-sectional study was conducted among 108 semi-professional football players. Dietary intake of simple sugars was assessed using validated dietary assessment tools, while serum levels of biochemical variables were measured using standard laboratory assays. Multinomial logistic regression analysis and partial correlation analysis were performed to examine the associations between dietary simple sugars and serum biomarkers, adjusting for confounders.

**Results:**

Strong positive associations were observed between dietary total simple sugar intake and hs-CRP and ADA levels in multinomial regression analysis. Also, among individual assessment of dietary simple sugars, dietary fructose and glucose intake were positively correlated with serum hs-CRP levels (*r* = 0.484, *P* < 0.001 and *r* = 0.393, *P* < 0.001, respectively) and serum ADA levels (*r* = 0.233, *P* = 0.001 for glucose; *r* = 0.188, *P* = 0.01 for fructose). There was no other association between dietary simple sugar intake and metabolic parameters.

**Conclusion:**

Our findings highlight the significant impact of dietary sugar intake on inflammation, as reflected by serum hs-CRP and ADA levels. Strategies aimed at reducing sugar consumption may help mitigate inflammation and improve overall health outcomes. Further research is warranted to elucidate the underlying mechanisms and to explore potential therapeutic interventions targeting dietary sugar intake for the prevention and management of chronic diseases.

## Popular scientific summary

- In the current study, dietary sugar intake was associated with increased inflammatory parameters including C-reactive Protein and adenosine deaminase levels.
- Reducing simple sugar consumption is recommended to reduce inflammation and improve overall health outcomes.

Nutrition plays a paramount role in sport, serving as the cornerstone of athletic performance, recovery, and overall well-being. Optimal nutrition provides the essential fuel needed to power through rigorous training sessions, competitions, and the demanding physical demands of sport (1). Beyond mere energy provision, the composition of an athlete’s diet influences critical physiological processes, including muscle repair and growth, hydration status, immune function, and cognitive performance (2, 3).

Among the various components of diet, the consumption of carbohydrate particularly simple sugars has emerged as a topic of interest, particularly in the realm of sports nutrition (4–6); semi-professional football players, in their quest for peak performance and injury prevention, often seek strategies to optimize their dietary intake.

It is essential for athletes to monitor their dietary intakes of carbohydrate to maintain and replenish muscle glycogen reserve; therefore, carbohydrate-rich diets are always considered as important source for endurance and ultra-endurance exercise because they are associated with increased muscle glycogen stores (7). However, the impact of dietary carbohydrate on athletic performance depends mostly on the type and amount of carbohydrate ingested (8); dietary simple carbohydrates including total intakes of disaccharides (sucrose, galactose, and lactose), monosaccharides (fructose, glucose, and maltose), and the content of added sugar in foods are readily available in many commonly consumed foods and beverages (9). Glucose is the primary fuel for human cells, and its polymers have been recommended as the predominant source of carbohydrates around endurance exercise sessions for athletes (10). On the other hand, fructose has been considered as a suboptimal source of carbohydrate to ingest during exercise due to its low effect on increasing exogenous carbohydrate oxidation and causing gastrointestinal distress (11).

Recently, the association between simple sugars intake and their health concerns attracted much attention; although dietary simple sugars provide a quick source of energy, excessive intake has been linked to various health concerns, including metabolic disorders, inflammation, and increased susceptibility to injury; Fajstova et al. demonstrated that a diet high in simple sugars increased gut permeability, promoted neutrophil infiltration, and increased the levels of interleukin (IL)-6, IL-1β, and tumor necrosis factor (TNF)-α via alterations in gut microbiota and toll-like receptor 4 signaling pathway (12). In other population-based studies, higher dietary simple and total sugar intake was associated with increased risk of metabolic syndrome (9, 13). Numerous studies also have revealed the role of dietary fructose in promotion of inflammation via pentose phosphate pathway (14) and increased serum pro-inflammatory cytokines (IL-6, TNF-α, and macrophage inflammatory protein-2) via gut microbiota alterations (15). Also, it has been shown that high-sugar diet promotes muscle injury via development of skeletal muscle insulin resistance and inflammation by stimulation of peroxisome proliferator-activated receptors (PPARs)-δ receptor agonism role (16).

In the intense physical activity, adenosine deaminase activity (ADA) plays an important role by direct inactivation of adenosine as an irreversible deamination of adenosine; ADA increases in serum as a result of muscle injury and also plays a potent inflammatory role in blood (17, 18). In the study by Chielle et al. (17), among 27 football players, ADA has been introduced as a promising novel biomarker of muscle injury increased after rigorous physical activity.

In the context of semi-professional football, where rigorous training regimens and intense physical exertion are routine, the impact of dietary choices on inflammatory processes and injury risk becomes particularly pertinent. Research exploring the relationship between dietary simple sugar intake and markers of inflammation and injury among semi-professional football players is not available. By elucidating this relationship, valuable insights can be gained into the potential mechanisms through which dietary factors influence athletic performance and recovery. Furthermore, such findings have the potential to inform targeted nutritional strategies aimed at optimizing performance and mitigating injury risk in this population. In the current cross-sectional study, we aim to investigate the association between dietary simple sugar intake, markers of inflammation, and injury among semi-professional football players.

## Methods

### Sampling procedure

The participants were recruited using a stratified random sampling technique from semi-professional football clubs within Ningb, China. Stratification was based on age (18–25 years and 26–35 years) and playing position (e.g. forward, midfielder, defender, and goalkeeper) to ensure representation across demographic and positional categories. Club administrators and coaches assisted in identifying eligible participants from their respective teams. Recruitment efforts involved informational sessions held at participating football clubs, during which potential participants were briefed on the study objectives, procedures, and eligibility criteria. Interested individuals were provided with detailed information sheets and invited to participate voluntarily. A written-informed consent was obtained from all participants prior to enrollment in the study.

### Sample size calculation

Sample size calculation was based on detecting a clinically significant difference in inflammatory markers between high and low dietary simple sugar intake groups, using previous study (13), alpha level of 0.05, and a power of 80%. Using G*Power software (Version 3.1.9.4), a total sample size of 97 participants was determined to achieve adequate statistical power for the planned analyses. Considering 10% of drop-out, a total of 108 participants were enrolled.

### Inclusion and exclusion criteria

Participants were required to meet the following inclusion criteria: Age between 18 and 35 years, active participation in semi-professional football, absence of any acute illness or injury at the time of enrollment, and willingness to provide informed consent for study participation. Individuals meeting any history of chronic inflammatory conditions (e.g. rheumatoid arthritis, inflammatory bowel disease, etc.), known metabolic disorders affecting glucose metabolism (e.g. diabetes mellitus), use of corticosteroids or other medications known to influence inflammatory markers, and inability or unwillingness to comply with study procedures were excluded.

### Dietary assessments

Participants completed a previously validated Food Frequency Questionnaire (FFQ) to assess their habitual dietary intake over a specified period (19). The FFQ consisted of a comprehensive list of foods and beverages. For each item, participants indicated their frequency of consumption (e.g. servings per day, week, or month) and portion size relative to standard references (e.g. small, medium, and large). Responses from the FFQ were processed and analyzed using specialized nutritional software (NutriSurvey 7.1) to estimate participants’ daily intake of key nutrients, including total energy, macronutrients (carbohydrates, proteins, and fats), micronutrients (vitamins and minerals), and dietary fiber. Simple sugar intake, including glucose, fructose, galactose, sucrose, lactose, and maltose, was calculated based on the reported consumption of sugary foods and beverages.

### Anthropometric assays and body composition analysis

Participants’ body weight and height were measured using a calibrated digital scale to the nearest 0.1 kg. Prior to measurement, participants were instructed to remove any heavy outer clothing and shoes, and to stand barefoot on the scale platform with their feet together and arms relaxed at their sides. Weight measurement was conducted in the morning, preferably after voiding, to minimize variations due to hydration status and food intake. Standing height was measured using a stadiometer to the nearest 0.1 cm. Participants stood erect with their back against the stadiometer, heels together, and head positioned in the Frankfurt plane. Body Mass Index (BMI) was calculated using the formula: BMI = weight (kg)/height squared (m). Waist circumference (WC) was measured at the midpoint between the lower rib margin and the iliac crest, in a horizontal plane, using a flexible, non-stretchable tape measuring to the nearest 0.1 cm. Bioelectrical Impedance Analysis (BIA) measures were used for body composition analysis by providing information about fat-free mass (FFM) (muscle, bone, and organs) and body fat percentage. Body composition was assessed using BIA (Inbody 770 Co., Seoul, Korea), following the manufacturer’s guidelines. Measurements were conducted when participants were in a fasting state and had emptied their bladders. Participants were instructed to remove any metal objects or jewelry and abstain from consuming caffeinated beverages and spices for at least 12 h prior to the measurements (20).

### Physical activity measurement

Participants completed self-report-validated International Physical Activity Questionnaire for Chinese adults (21) to provide information on the frequency, duration, and intensity of their physical activity across various domains (e.g. leisure-time, occupational, and transportation). These questionnaires included items prompting participants to recall their activities over a specified time frame, typically the past week or month.

### Biochemical assessments

Blood samples were collected following an overnight period of fasting. Serum lipid levels, which encompassed total cholesterol (TC), high-density lipoprotein cholesterol (HDL-C), low-density lipoprotein cholesterol (LDL-C), and triglycerides (TGs), were assessed using standard laboratory assays conducted with an automated analyzer (Alpha Classic E analyzer). Serum insulin levels were determined utilizing commercial kits (AccuBind, Insulin, USA, Monobind Inc.). Serum high-sensitivity C-reactive protein (hs-CRP) concentrations were quantified through an enzymatic immunoassay turbidimetric method (Roche Cobas 6000, Penzberg, Germany). Serum ADA was assessed using a commercial ELISA kit (Elabscience; USA), with a sensitivity of 0.03 U/L.

### Statistical analysis

Statistical analysis was performed using appropriate software (e.g. SPSS, version 23). Descriptive statistics were used to summarize participant characteristics, dietary intake, and biochemical markers. Analysis of variance was used to compare the variables between tertiles of dietary simple sugar intake. Bivariate correlation analysis (e.g. Partial correlation coefficient) was employed to examine the associations between dietary individual simple sugar intake and inflammation and injury markers. Multiple regression analysis was utilized to assess the independent contribution of dietary factors to metabolic and inflammatory parameters, adjusting for potential confounders, such as age, sex, BMI, and physical activity level. *P*-values less than 0.05 were considered as statistically significant.

## Results

**Fig. 1:**
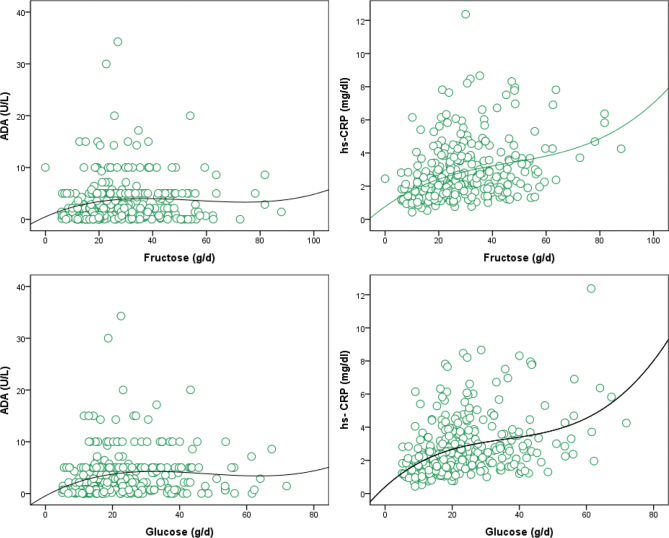
Partial correlation analysis of the associations between dietary fructose and glucose with serum CRP and ADA levels after adjustment for confounders (e.g. age, sex, and physical activity). The associations were strong for with dietary glucose and fructose and serum hs-CRP levels (*r* = 0.484; *P* < 0.001 and *r* = 0.393; *P* < 0.001, respectively) while were modest for the associations between dietary glucose and fructose and serum ADA levels (*r* = 0.233; *P* = 0.001 and *r* = 0.188; *P* = 0.01, respectively).

Table 1 illustrates the general demographic characteristics of the study participants categorized by tertiles of total dietary simple sugar intake. The total number of participants included in this study was 108. The mean age of participants across the tertiles ranged from 28.74 to 32.44 years, with a statistically significant difference observed (*P* = 0.035*). The distribution of males across the tertiles was relatively balanced, with percentages ranging from 47.22 to 52.77% (*P* = 0.98). No significant differences were found in BMI, WC, fat mass (FM), FFM, or PA across the tertiles (*P* > 0.05). Table 2 presents the individual dietary intake of simple sugars among study participants categorized by tertiles of total dietary simple sugar intake. Statistically significant differences were observed in the mean intake of all simple sugars across the tertiles (*P* < 0.001), indicating variations in dietary sugar consumption among the groups as expected. Specifically, as the tertile of total dietary simple sugar intake increased, the mean intake of glucose, galactose, fructose, saccharose, lactose, and maltose also increased. Table 3 displays the dietary intake of energy, macronutrients, and food groups among study participants, stratified by tertiles of total dietary simple sugar intake. Statistically significant differences were observed in the mean intake of energy, macronutrients (carbohydrate, protein, and fat), and various food groups across the tertiles (*P* < 0.05), indicating variations in dietary patterns among the groups. Specifically, as the tertile of total dietary simple sugar intake increased, participants exhibited higher mean energy intake and a greater proportion of energy from carbohydrates, along with lower percentages of energy from protein and fat. Additionally, significant differences were observed in the intake of food groups such as grains, legumes, high-fat dairy, low-fat dairy, vegetables, fruit, and nuts across the tertiles. Table 4 presents the association between biochemical variables and total dietary simple sugar intake among study participants. The table displays odds ratios (ORs) and their corresponding confidence intervals (CIs) for each blood biomarker across the tertiles of simple sugar intake. For each biomarker, three models were examined: Model I (crude), Model II (adjusted for age and sex), and Model III (adjusted for age, BMI, sex, and physical activity). The results indicate varying associations between simple sugar intake and blood biomarkers across the different models. Notably, significant associations were observed between simple sugar intake and hs-CRP in models II and III and for the association between simple sugar intake and ADA levels in all of three models, with *P*-values < 0.05. There was no association between dietary simple sugar intake and other biochemical parameters. Figure 1 illustrates the results of partial correlation analysis examining the associations between dietary fructose and glucose intake with serum CRP and ADA levels, after adjusting for confounding factors such as age, sex, and physical activity. The analysis revealed strong associations between dietary glucose and fructose intake with serum hs-CRP levels, with correlation coefficients (*r*) of 0.484 (*P* < 0.001) and 0.393 (*P* < 0.001), respectively. Conversely, the associations between dietary glucose and fructose intake with serum ADA levels were more modest, with correlation coefficients of 0.233 (*P* = 0.001) and 0.188 (*P* = 0.01), respectively. The association between dietary intake of other individual sugars with hs-CRP or ADA was weak or no association was found.

**Table 1:** General demographic characteristics of study participants by tertiles of total simple sugar intake

Parameter	Total (*n* = 108)		Tertiles of total dietary simple sugar						*P* *
			T_1_ (*n* = 36)		T_2_ (*n* = 36)		T_3_ (*n* = 36)		
	Mean	SD	Mean	SD	Mean	SD	Mean	SD	
Age (year)	30.62	9.19	28.85	9.42	29.56	7.78	32.44	10.01	0.035*
Sex (% male)	54	50	18	50	17	47.22	19	52.77	0.98**
BMI (kg/m^2^)	28.68	4.81	28.74	4.32	29.00	5.14	28.28	4.97	0.526*
WC (cm)	96.69	9.59	95.46	9.16	93.39	9.53	99.23	10.12	0.633*
FM (%)	33.81	9.13	33.82	7.47	33.86	11.31	33.72	8.20	0.997*
FFM (%)	62.25	12.35	60.86	12.98	63.68	12.38	62.31	11.43	0.413*
PA (Met.min/week)	2649.47	328.12	2047.56	332.41	2652.72	437.95	3248.15	450.4	0.312*

All data are mean (±SD) except sex, that is presented as the number and percent.

P* values derived from One-Way ANOVA with Tukey’s post-hoc comparisons.

** *P*-values derived from chi-squared test.

Bold P-values are statistically significant.

**Table 2:** Individual dietary intake of simple sugars of study participants by tertiles of total simple sugar intake

Variable	Tertiles of total dietary simple sugar						*P* *
	T_1_ (*n* = 36)		T_1_ (*n* = 36)		T_1_ (*n* = 36)		
	Mean	SD	Mean	SD	Mean	SD	
Glucose (g/day)	13.92	5.17	20.92	5.42	35.15	12.62	**<0.001**
Galactose (g/day)	1.48	1.33	2.44	1.79	4.22	4.21	**<0.001**
Fructose (g/day)	16.73	5.67	25.56	6.88	42.32	14.69	**<0.001**
Saccharose (g/day)	20.68	8.81	38.88	12.61	74.06	14.14	**<0.001**
Lactose (g/day)	7.75	5.36	14.39	9.39	19.64	12.39	**<0.001**
Maltose (g/day)	2.19	1.18	2.60	1.61	3.32	2.02	**<0.001**

* *P*-values derived from One-Way ANOVA or Kruskal-Wallis test where needed.

Bold P-values are statistically significant.

**Table 3:** Dietary intake energy, macronutrients, and food groups of participants according to tertiles of simple sugar intake

Variable	Tertiles of total dietary simple sugar						*P* *
	T_1_ (*n* = 36)		T_1_ (*n* = 36)		T_1_ (*n* = 36)		
	Mean	SD	Mean	SD	Mean	SD	
Energy (kcal/d)	2344.68	699.58	2891.39	758.06	3822.36	1195.68	**<0.001**
Carbohydrate (%)	57.75	7.11	59.43	6.57	62.99	7.44	**<0.001**
Protein (%)	13.57	2.17	13.76	2.21	12.79	2.04	**0.002**
Fat (%)	30.91	7.33	29.91	6.18	28.08	6.47	**0.006**
Grains (g/day)	539.97	236.27	557.35	221.80	626.92	315.57	0.032*
Legumes (g/day)	45.99	35.03	57.81	50.99	69.23	77.29	0.010*
Red meat (g/day)	29.89	33.44	33.64	31.46	36.45	35.04	0.338*
Poultry (g/day)	25.65	23.26	29.55	34.54	32.52	27.28	0.201*
Fish (g/day)	8.19	12.32	12.08	17.16	10.41	11.03	0.106*
High fat dairy (g/day)	62.23	80.14	117.28	136.32	179.62	210.34	<0.001*
Low fat dairy (g/day)	155.49	110.29	274.98	191.81	355.28	239.36	<0.001*
Vegetables (g/day)	243.64	154.09	338.26	168.72	442.46	344.23	<0.001*
Fruit (g/day)	357.52	342.52	579.05	307.52	1196.87	643.07	<0.001*
Nuts (g/day)	12.10	20.55	18.40	39.26	24.07	56.70	0.098*

P* values derived from energy-adjusted ANCOVA except for dietary energy intake.

Bold P-values are statistically significant.

**Table 4:** Association between biochemical variables and simple sugar intake among participants

Blood biomarker		Tertiles of total dietary simple sugar				
		T_1_	T_2_		T_3_	
			OR (CI)	*P*	OR (CI)	*P*
LDL (mg/dL)	Model I	**1** **REF**	0.994 (0.986–1.003)	0.195	1.002 (0.994–1.011)	0.565
	Model II		0.994 (0.986–1.003)	0.195	1.002 (0.994–1.010)	0.674
	Model III		0.990 (0.978–1.002)	0.114	0.996 (0.981–1.012)	0.631
HDL (mg/dL)	Model I	**1** **REF**	1.008 (0.981–1.037)	0.551	1.002 (0.974-1.030)	0.895
	Model II		1.013 (0.984–1.042)	0.399	1.003 (0.974–1.033)	0.851
	Model III		1.039 (0.995–1.085)	0.082	0.987 (0.932–1.045)	0.655
TG (mg/dL)	Model I	**1** **REF**	1.000 (0.998–1.003)	0.752	1.001 (0.999–1.004)	0.330
	Model II		1.000 (0.997–1.003)	0.824	1.001 (0.998–1.004)	0.462
	Model III		0.999 (0.992–1.006)	0.780	1.002 (0.994–1.011)	0.601
TC (mg/dL)	Model I	**1** **REF**	0.996 (0.989–1.003)	0.269	1.001 (0.994–1.008)	0.786
	Model II		0.996 (0.989–1.003)	0.272	1.000 (0.993–1.007)	0.978
	Model III		0.994 (0.983–1.005)	0.270	0.997 (0.983–1.012)	0.715
FBS (mg/dL)	Model I	**1** **REF**	1.008 (0.991–1.025)	0.362	1.012 (0.996–1.028)	0.151
	Model II		1.008 (0.991–1.025)	0.359	1.010 (0.994–1.026)	0.222
	Model III		1.006 (0.981–1.032)	0.643	1.017 (0.990–1.044)	0.219
Insulin (mIU/L)	Model I	**1** **REF**	0.992 (0.971–1.014)	0.487	0.989 (0.965–1.013)	0.360
	Model II		0.990 (0.968–1.013)	0.385	0.983 (0.959–1.007)	0.167
	Model III		1.037 (0.995–1.080)	0.086	1.027 (0.973–1.084)	0.334
CRP (mg/dL)	Model I	**1** **REF**	1.25 (1.027–1.53)	0.027	1.219 (0.97–1.514)	0.083
	Model II		1.25 (1.026–1.54)	0.028	1.61 (1.32–1.97)	**<0.001**
	Model III		1.57 (1.29–1.94)	<0.001	1.46 (1.16–1.83)	**<0.001**
ADA (U/L)	Model I	**1** **REF**	1.071 (0.99–1.14)	0.053	1.104 (1.03–1.18)	**0.004**
	Model II		1.074 (1.00–1.152)	0.048	1.036 (1.005–1.067)	**0.003**
	Model III		1.143 (1.025–1.27)	0.017	1.22 (1.093–1.36)	**<0.001**

The multinomial logistic regression was used for estimation of ORs and confidence interval (CI). Model I: crude, Model II: adjusted for age and sex, Model III: adjusted for age, BMI, sex, and physical activity.

Bold P-values are statistically significant.

## Discussion

The present study investigated the associations between dietary simple sugar intake and serum metabolic and inflammatory biomarkers among semi-professional football players. The findings reveal notable correlations between total dietary sugar intake and markers of inflammation, highlighting the potential impact of diet on health outcomes. No association was found between dietary simple sugar intake with metabolic or glycemic markers. Moreover, among dietary simple sugars, the strongest association was attributed to dietary fructose and glucose intakes. The strong positive associations observed between dietary fructose and glucose intake with serum hs-CRP levels suggest a significant relationship between sugar consumption and systemic inflammation. Previous research has implicated high sugar intake, particularly fructose, in the development of low-grade chronic inflammation (4, 9, 14, 15), which is a key contributor to various chronic diseases such as obesity, type 2 diabetes, and cardiovascular disease. The pro-inflammatory effects of sugars may be attributed to several mechanisms, including increased production of pro-inflammatory cytokines, oxidative stress, and activation of inflammatory pathways such as the nuclear factor-kappa B pathway (22). Increased gut permeability, promoted neutrophil infiltration, and increased levels of IL-6, IL-1β, and TNF-α (12), and increased skeletal muscle insulin resistance and inflammation via simulating PPAR-δ (16) are some suggested mechanisms attributed to simple sugar intake. Conversely, the associations between dietary fructose and glucose intake with serum ADA levels were more modest. ADA is an enzyme involved in purine metabolism and has been suggested as a marker of immune function and activation. Although it is suggested also as a potent pro-inflammatory parameter (23), the weaker correlations observed in our study may suggest a less direct influence of dietary sugars on immune activity as reflected by ADA levels. However, there are very limited number of studies, and further research is needed to elucidate the specific mechanisms underlying the relationship between dietary sugars and ADA levels. The findings underscore the importance of dietary sugar intake in modulating inflammation and immune responses, with potential implications for the prevention and management of chronic diseases. Strategies aimed at reducing sugar consumption, particularly sources of added sugars such as sugary beverages, processed foods, and desserts, may help mitigate inflammation and improve overall health outcomes. Additionally, promoting a balanced diet rich in whole foods, fruits, vegetables, and lean proteins may help attenuate the inflammatory effects of high sugar intake (24, 25). Limitations include its cross-sectional design, which precludes establishment of causality, and the reliance on self-reported dietary intake data, which may be subject to recall bias. Future research incorporating longitudinal studies and objective measures of dietary intake and biomarkers is warranted to further elucidate the causal relationships between dietary sugars and inflammation/immune function.

For future directions, it is better to assess other oxidative stress biomarkers, including malondialdehyde and other lipid peroxidation byproducts to provide valuable insights into oxidative damage within biological systems. Additionally, the investigation of the effect of dietary simple sugar intake on the regulation of stress hormones, specifically cortisol and adrenaline, is suggested to provide a clearer understanding of the metabolic consequences associated with sugar consumption and its potential links to stress and overall health.

In conclusion, our study provides valuable insights into the associations between dietary fructose and glucose intake and serum levels of hs-CRP and ADA. These findings underscore the importance of dietary sugar reduction as a potential strategy for mitigating inflammation and improving immune function, ultimately contributing to better overall health outcomes among young semi-professional athletes.
